# Effect of Fibril Entanglement on Pickering Emulsions Stabilized by Whey Protein Fibrils for Nobiletin Delivery

**DOI:** 10.3390/foods11111626

**Published:** 2022-05-31

**Authors:** Fangcheng Jiang, Chunling Chen, Xinlan Wang, Wenjing Huang, Weiping Jin, Qingrong Huang

**Affiliations:** 1 Key Laboratory for Deep Processing of Major Grain and Oil, School of Food Science and Engineering, Wuhan Polytechnic University, Wuhan 430023, China; fj109@scarletmail.rutgers.edu (F.J.); ccl3529151143@163.com (C.C.); wxl19980609@163.com (X.W.); huangwenjingbest@163.com (W.H.); 2 Department of Food Science, Rutgers University, 65 Dudley Road, New Brunswick, NJ 08901, USA

**Keywords:** whey protein isolate, Pickering emulsion, fibrils, interfacial structure, overlap concentration, bioaccessibility, nobiletin

## Abstract

The aim of the study was to investigate the effects of whey protein isolate (WPI) fibrils entanglement on the stability and loading capacity of WPI fibrils-stabilized Pickering emulsion. The results of rheology and small-angle X-ray scattering (SAXS) showed the overlap concentration (C*) of WPI fibrils was around 0.5 wt.%. When the concentration was higher than C*, the fibrils became compact and entangled in solution due to a small cross-sectional radius of gyration value (1.18 nm). The interfacial behavior was evaluated by interfacial adsorption and confocal laser scanning microscopy (CLSM). As the fibril concentration increased from 0.1 wt.% to 1.25 wt.%, faster adsorption kinetics (from 0.13 to 0.21) and lower interfacial tension (from 11.85 mN/m to 10.34 mN/m) were achieved. CLSM results showed that WPI fibrils can effectively absorb on the surface of oil droplets. Finally, the microstructure and in vitro lipolysis were used to evaluate the effect of fibrils entanglement on the stability of emulsion and bioaccessibility of nobiletin. At C* concentration, WPI fibrils-stabilized Pickering emulsions exhibited excellent long-term stability and were also stable at various pHs (2.0–7.0) and ionic strengths (0–200 mM). WPI fibrils-stabilized Pickering emulsions after loading nobiletin remained stable, and in vitro digestion showed that these Pickering emulsions could significantly improve the extent of lipolysis (from 36% to 49%) and nobiletin bioaccessibility (21.9% to 62.5%). This study could provide new insight into the fabrication of food-grade Pickering emulsion with good nutraceutical protection.

## 1. Introduction

Pickering emulsions have been widely applied in foods, pharmaceuticals, and cosmetics due to their outstanding characteristics, such as extraordinary stability, controlled release of components, and targeted nutraceutical delivery [1,2,3]. The formation and stability mechanisms of Pickering emulsions are affected by some factors, such as particle concentration, oil fraction, pH, ionic strength, and temperature [4,5,6].

The particle concentration is one of the most important and easily adjusted factors during Pickering emulsion formation [7]. In general, droplet size decreases with increasing particle concentration (at a fixed oil-to-water ratio) until it reaches a plateau level. This effect is attributed to two factors: (1) more particles are available to cover the oil droplet surfaces with concentration increasing; (2) sufficient particles cover all the oil droplet surfaces against re-coalescence [8]. The droplet sizes of emulsion also play an important role in emulsion stability due to the lower interfacial layer caused by large droplet sizes [9]. Feng et al. reported that the apparent viscosity and modulus, and stability of food-grade gelatin nanoparticle–stabilized Pickering emulsions were improved by increasing the nanoparticle concentration [7]. Gao et al. found that Pickering emulsions stabilized by protein fibrils showed excellent physical stability at appropriate fibril concentrations (0.5–2.0%), while excessive fibril concentrations (≥2.5%) resulted in larger emulsion droplet size [10]. This is attributed to the accumulation and aggregation of excess particles in the bulk phase. The aggregation not only increases the particle size but also forms a network on the surface of emulsion droplets or a gel-like structure in the continuous phase. When the particle concentration of pea protein isolate (PPI) nanoparticles is larger than 2 g/100 mL, the gel-like whole emulsion without creaming is obtained [11,12]. In addition, particle concentration plays a key role in the dynamic adsorption at the oil–water interface, which can further affect the properties of Pickering emulsions and their applications [13]. It was reported that the diffusion at the interface is a concentration-dependent process, and the emulsification performance of the nanoparticles and the stability of the corresponding emulsions were improved at high concentrations (>0.5%, *w*/*v*) [14]. Although the effect of particle concentration on Pickering emulsions has attracted much attention in recent years, few works have focused on fibril, especially if the fibril concentration is higher than overlap concentration (C*) in solutions.

Whey protein isolate (WPI), a byproduct of the cheese-making process, comprises proteins such as β-lactoglobulin, α-lactalbumin, and bovine serum albumin [15,16]. WPI could form fibrils through heat processing at acidic pH and low ionic strength [17,18]. These fibrils are usually a few nanometers in diameter but up to several micrometers in length, and the phase behavior in solution is similar to that of polymers. The critical overlap concentration (C*) is the intermediate state of the polymer between the dilute solution and the semi-concentrated solution. When the polymer concentration is lower than C*, the molecules move freely in the solution, while if the polymer concentration is higher than C*, molecules begin to interact and entangle [19]. In applications, WPI fibrils are potential emulsifiers and have been used to deliver lipophilic ingredients [20,21,22]. The effect of the C* of WPI fibrils on the oil-water interfacial behavior, stability of emulsions, and delivery efficiency has not been confirmed.

Nobiletin, a poly-methoxylated flavone (PMF) isolated from the peels of various citrus fruits, is a model for lipophilic ingredients [23,24]. It has lots of potential health benefits, but the poor water solubility (~0.9 μM in the aqueous phase) and low bioaccessibility limit nobiletin applications in the food industry [24,25]. This work aims to study the influence of concentration on the structural and interfacial properties of WPI fibrils. Then, Pickering emulsions stabilized by WPI fibrils were prepared to evaluate the in vitro bioaccessibility of nobiletin and to understand the relationship between interfacial structure and performance of WPI fibril-stabilized Pickering emulsions in delivering lipophilic nutraceuticals.

## 2. Materials and Methods

### 2.1. Materials

Whey protein isolate (WPI, Hilmar™ 9020) (protein content of 89.5%) was purchased from Hilmar Company (Hilmar Cheese, CA, USA). Soybean oil was supplied by Yihai Kerry Arawana Oils and Grains Industries Ltd. (Shanghai, China). Nobiletin (HPLC purity 98%) was obtained from Sichuan Weikeqi Biological Technology, Co., (Chengdu, China). Pancreatin from porcine pancreas and pepsin from porcine gastric mucosa were purchased from Sigma-Aldrich (St. Louis, MO, USA). All solutions were prepared with Millipore water (Millipore, MA, USA). All other chemical reagents, including thioflavin T (ThT), hydrogen chloride (HCl), and sodium hydroxide (NaOH), were analytical grade.

### 2.2. Preparation of WPI Fibrils

WPI fibrils were prepared according to our previous study [6]. Briefly, 2 wt.% WPI solution was adjusted to pH 2.0 and heated at 90 °C for 5 h. Non-fibril peptides were removed via dialysis with pH 2.0 water for three days. WPI fibrils were dispersed in water at various concentrations (from 0.1 wt.% to 1.25 wt.%).

### 2.3. Rheology Analysis

The viscosity of WPI fibrils was measured by a rheometer (Malvern Panalytical Limited, Malvern, England, UK) using a 60 mm cone and plate, with a shear rate ranging from 0.01 to 1000 s^−1^. All samples were measured at 25 °C. The obtained viscosity, as a function of shear rate data, was fitted to Cross models (Equation (1)) to obtain the relevant parameters [26]. The shape and curvature of a flow curve can be described through this model and the behavior at unmeasured shear rates can be predicted as well.

(1) $$\frac{\eta -\eta_{\infty }}{\eta_{0}-\eta_{\infty }}=\frac{1}{1+{\left(K\dot{\gamma }\right)}^{m}}$$

where η_0_ is the zero-shear viscosity; η_∞_ is the infinite shear viscosity; K is the cross constant, which is indicative of the onset of shear-thinning; $\dot{\gamma }\;$ is the shear rate or strain rate; and *m* is the shear thinning index, which ranges from 0 (Newtonian) to 1 (infinitely shear thinning).

### 2.4. SAXS Analysis

The samples were prepared with a series of WPI fibril concentrations (0.1, 0.25, 0.5, 1.0, 1.25 wt.%). A detector located 2631 mm from the sample was set to collect the scattering intensity, and the wavelength of X-ray radiation was 1.033 Å. The final scattering data were obtained by averaging 20 curves, with the scattering vector Q ranging from 1.0 × 10^−2^ to 4.1 × 10^−1^ Å^−1^.

### 2.5. Dilatational Interfacial Rheology

The change in the interface tension (mNm^−1^) at the oil–water interface with adsorption time (t) was measured by droplet shape analysis (DSA30S, KRUSS, Hamburg, Germany). The WPI fibril solution was collected in a syringe with a diameter of 1.8 mm. The needle tip was immersed in a rectangular glass tank filled with soybean oil. The interfacial tension value was recorded at 1 frame/s for the first 1 h and 0.1 frame/min thereafter, and the value was monitored continuously for 10,800 s. All measurements were performed at 25 °C.

### 2.6. Preparation of WPI Fibril-Stabilized Pickering Emulsion with and without Nobiletin

#### 2.6.1. Preparation of Pickering Emulsions Stabilized by WPI Fibrils

Samples were mixed with different WPI fibril concentrations (0.1–1.25 wt.%) and a constant oil phase volume (φ = 50%). The samples were emulsified using a high-speed homogenizer at 10,000 rpm for 3 min (Ultra Turrax, T18 digital, IKA, Staufen, Germany).

#### 2.6.2. Preparation of Nobiletin-Loaded Pickering Emulsions Stabilized by WPI Fibrils

Nobiletin was dissolved in soybean oil by heating (100 °C for 1 h) in an oil bath to a final concentration of 0.5 wt.%. The nobiletin-loaded Pickering emulsion was prepared as described above.

### 2.7. Characterization of WPI Fibril-Stabilized Pickering Emulsion

#### Confocal Laser Scanning Microscopy

Confocal laser scanning microscopy (FV1200, Olympus, Tokyo, Japan) was performed to confirm the formation of Pickering emulsions. Prior to confocal laser scanning microscopy analysis, samples were dyed by ThT. Images were obtained using a 20× magnification lens at an excitation wavelength of 488 nm. ImageJ software was used to estimate the mean droplet diameters.

### 2.8. Optical Microscopy

The microstructure images of the Pickering emulsion stabilized by WPI fibrils were obtained using an optical microscope (CX40, Sunny Optical Technology Co., Ltd., Yuyao, China) equipped with a camera. Thirty microliters of Pickering emulsion were deposited on the glass slide and covered with a coverslip. All samples were measured with a magnification of 20× at room temperature. Nobiletin-loaded Pickering emulsions were characterized using the same method described for the unloaded Pickering emulsions.

### 2.9. Physicochemical Stability of WPI Fibril- Stabilized Pickering Emulsions

#### 2.9.1. Effect of pH

Freshly prepared samples of Pickering emulsions were diluted 10 times using de-ionized water, and the pH was adjusted to pH 2.0, 3.0, 4.0, 5.0, 6.0, and 7.0, respectively, with 0.1 M NaOH solutions.

#### 2.9.2. Effect of Ionic Strength

Freshly prepared samples of Pickering emulsions were diluted 10 times using 0 mM to 200 mM NaCl solutions.

#### 2.9.3. Effect of Storage Time

After the preparation of emulsions, the freshly prepared sample emulsions were stored at room temperature. The microstructure of the emulsions was determined at regular storage periods (1, 7, and 28 d).

### 2.10. In Vitro Digestion Analysis, Free Fatty Acid Release, and Bioaccessibility of Nobiletin

#### 2.10.1. Digestion of Pickering Emulsions Stabilized by WPI Fibrils

This study used an in vitro model consisting of oral, gastric, and intestine phases slightly modified from Brodkorb et al. [27]. Salivary amylase was not added in the oral digestion phase in this study. Fresh emulsions were prepared with an oil fraction of 50% (*w*/*w*). The samples, including soybean oil and WPI fibril-stabilized Pickering emulsion containing 0.25 g of oil, were mixed with 4 mL of simulated salivary fluid (SSF), 0.025 mL of 0.3 M CaCl_2_, and 0.225 mL of pure water for 2 min. Eight milliliters of simulated gastric fluid (SGF), 0.005 mL of 0.3 M CaCl_2_, 0.4 mL of 5 M HCl, and 0.448 mL of pure water were added to oral digestive juice successively, and freshly dissolved pepsin was added to achieve enzyme activity of 2000 U/mL and to initiate the gastric digestion process. Subsequently, the reaction mixture was incubated under continuous stirring in a temperature-controlled oil bath (37.0 ± 0.1 °C) for 2 h. Eight milliliters of simulated intestinal fluid (SIF), 0.04 mL of 0.3 M CaCl_2_, 10 mM bile salt, and 3.16 mL of pure water were added to the gastric digesta, and the pH was adjusted to 7.0 with the addition of NaOH. Pancreatin was added to gastric digesta to obtain an enzyme activity of 100 U/mL to start intestinal digestion. The mixture was incubated under continuous agitation in an oil bath (37.0 ± 0.1 °C) for 2 h, and 0.1 M NaOH was added manually to maintain the pH at 7.0 during lipolysis. The volume of added NaOH solution was recorded over time during the intestinal digestion. Lipolysis in samples was characterized by the release of free fatty acids (**FFA**). The fraction of **FFA** released was calculated as follows:

(2) $$\%FFA=100\times \frac{M_{lipid}\times V_{\mathrm{NaOH}}\times m_{\mathrm{NaOH}}}{W_{lipid}\times 2}$$

where ***M*_lipid_** is the molecular mass of the triacylglycerol oil (in g/mol); ***V***_NaOH_ is the volume of NaOH solution used to neutralize the released **FFA** (in L); ***m***_NaOH_ is the molarity of NaOH solution (in mol/L); and ***W*_lipid_** is the total mass of the initial triacylglycerol oil. The molecular mass of soybean oil was 876.56 g/mol.

#### 2.10.2. High-Performance Liquid Chromatography (HPLC) Analysis of Nobiletin

The digests were collected after gastrointestinal digestion and centrifuged at 10,000× *g* for 40 min. The clear micelle phase was collected, and the nobiletin content in the micelle phase was determined by an UltiMate 3000 HPLC system (Dionex, Sunnyvale, CA, USA), with a SunFire C18 column (150 mm × 4.6 mm, 5 μm). The HPLC mobile phase consisted of (A) acetonitrile and (B) water. The elution conditions were as follows: 45% (A) and 55% (B), and the running time was 15 min. The detection wavelength was 333 nm, and the concentration of nobiletin was determined using a standard curve of nobiletin.

#### 2.10.3. Determination of Nobiletin Bioaccessibility

After HPLC quantification of nobiletin in the micelle phase, nobiletin bioaccessibility was determined by the following equation [28]:

(3) $$bioaccessibility=\frac{nobiletin\;content\;in\;the\;micelle\;phase}{total\;nobiletin\;content\;in\;the\;formulations}\times 100\%$$

### 2.11. Statistical Analysis

Each experiment was conducted in triplicate. All statistical analyses were performed using OriginPro 9.0. Duncan’s test of SPSS Statistics 26 was used, and significance was set at *p* < 0.05.

## 3. Results and Discussion

### 3.1. Rheology

**Figure 1:**
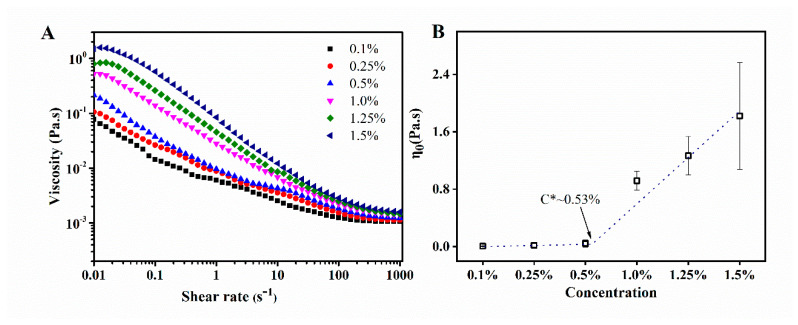
Rheology diagram of WPI fibrils solutions with the concentrations of 0.1–1.25 wt.% (**A**) Steady flow curves and (**B**) the relationship between η_0_ and of WPI fibrils concentrations (pH 2.0).

In polymer solution, there are three regimes: dilute, semi-dilute, and concentrated. The transition from the dilute to the semi-dilute regime depends on the concentration of the polymer solution, and entanglement occurs at concentrations above the overlap threshold (C*) [29]. The C* can be determined by measuring the concentration at which the viscosity of the polymer solution increases suddenly. Nanofibrils are, to some extent, similar to polymers, which have a few nanometers in diameter but up to several micrometers in length. As shown in Figure 1A, the viscosity of WPI fibril solutions at the same concentration decreased with increasing shear rates, indicating that all samples showed shear-thinning behavior, a type of non-Newtonian behavior. At the same shear rates, the viscosity of WPI fibrils increased with increasing WPI fibril concentration, which was attributed to molecule chain entanglement [26]. There was more entanglement among protein molecules as the WPI fibril concentration increased. In addition, at low enough shear rates, shear-thinning samples showed a constant viscosity, which was defined as the zero-shear viscosity (*η_0_*). Therefore, to further understand the steady flow behavior of WPI fibrils, the Cross model was applied in Figure 1B. At low concentrations (0.1–0.25 wt.%), the *η_0_* of the WPI fibrils did not change significantly, indicating that the WPI fibrils were flexible and moved freely in the solution. As the concentration increased to 0.5 wt.%, the *η_0_* increased abruptly, which indicated that it was a critical overlap concentration (C*) [26]. At this concentration, WPI fibrils were closer to each other, and entanglement occurred between the WPI fibrils. As the concentration increased further, the density of entanglement between the WPI fibrils increased, which led to an increase in the viscosity of the WPI fibrils.

### 3.2. SAXS

**Figure 2:**
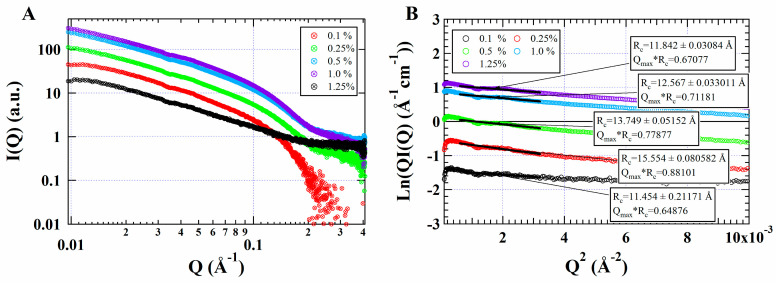
Small-angle X-ray scattering (SAXS) at different WPI fibrils concentration. (**A**) Profiles of SAXS intensity I(q) and (**B**) Guinner plots of WPI fibrils with different concentrations (0.1–1.25%) at pH 2.0.

Small-angle X-ray scattering (SAXS) is a powerful method that probes the structure of biomolecules at the nanoscale [30]. Figure 2A shows the scattering intensity profiles of WPI fibrils at various concentrations. In a previous study, β-Lg fibrils exhibited a rigid rod-like structure at low concentrations (0.2 and 0.3 wt.%) since the slope of the low Q region of the scattering profiles was −1 [6]. Guinier analysis is a straightforward approach to determining the cross-sectional radius of gyration (R_c_), and the shape of the primary nanostructure is calculated via Guinier fitting in the Q range of 0.03–0.05 Å [31]. Guinier plots of WPI fibrils are presented in Figure 2B. The R_c_ of WPI fibrils from 0.1 wt.% to 1.25 wt.% was 11.45 Å, 15.55 Å, 13.75 Å, 12.57 Å, and 11.84 Å, respectively. The value of R_c_ increased when WPI fibril concentration increased, and it decreased after the concentration exceeded 0.5 wt.%, which suggested that there were two distinct, concentration-dependent scaling regions in the R_c_ of WPI fibril solutions. These regions are analogous to the threshold between dilute and semi-dilute regions in WPI fibrils dissolved in isolated form. Moreover, there was hardly any intermolecular interaction when the concentration of WPI fibrils was lower than 0.5 wt.%. Above 0.5 wt.%, the fibril size decreased upon the increase in fibril concentration, which may be due to intermolecular interpenetration [32]. The threshold concentration between the two distinct scaling regions was very close to the C* of the WPI fibril solution.

### 3.3. Interfacial Adsorption Behavior

**Figure 3:**
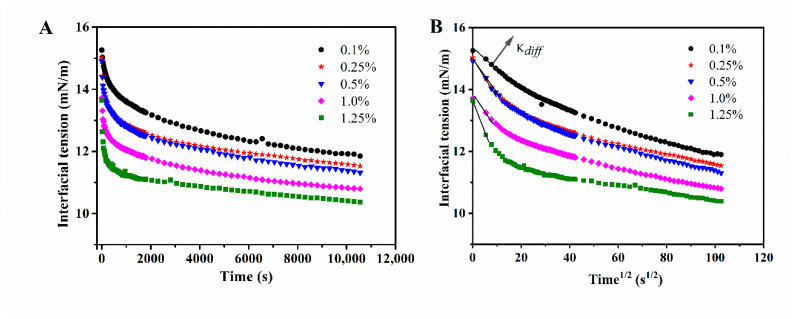
Interfacial behavior of WPI fibrils at the oil/water interface. (**A**) Time evolution of the interfacial tension (γ) for WPI fibrils (0.1–1.25 wt.%) at pH 2.0 and (**B**) dynamic interfacial tension vs. square root of time.

To evaluate the role of the WPI fibril concentration in the formation dynamics of the films at the oil–water interface, the interfacial adsorption behavior of the WPI fibrils in relation to concentration was investigated. Figure 3A shows the time evolution of interfacial tension for the WPI fibrils (0.1–1.25 wt.%) at the oil–water interface. Initially, the interfacial tension values of WPI fibrils with concentrations of 0.1–1.25 wt.% were 15.96 mN/m, 15.36 mN/m, 15.08 mN/m, 13.98 mN/m, and 13.47 mN/m, respectively. This suggested that a significant reduction in interfacial tension occurred when the concentration was higher than C*. The interfacial tension values of WPI fibrils rapidly decreased with adsorption time and exhibited a notable dependence on fibril concentration, which suggests a high concentration could positively promote the adsorption process [33]. The diffusion rate was used to explain the migration of proteins from the bulk phase to the oil–water interface [34]. *K*_diff_ is an estimation of the rate of initial diffusion-controlled migration, and it was dependent on the concentration in the bulk phase [35]. The diffusion of fibrils from the bulk phase to the interface occurred at relatively short adsorption times (up to about 47.8 s) [35], and gradually reached equilibrium [36]. Figure 3B shows that the *K*_diff_ values increased with increasing WPI fibril concentration. Thus, the rate of WPI adsorption was faster at higher concentrations. This suggests the concentration gradient is the driving force for the diffusion of WPI fibrils, which was supported by the previous results for whey protein isolate at the oil–water interfaces [35]. Liu et al. studied the diffusion of soy glycinin when the particle concentration was low (0.01 wt.%). While diffusion-controlled adsorption occurred at a high concentration (>0.5 wt.%) during the initial periods of adsorption, it can be inferred that diffusion is related to the C* [14].

### 3.4. Characterization of WPI Fibril-Stabilized Emulsions

**Figure 4:**
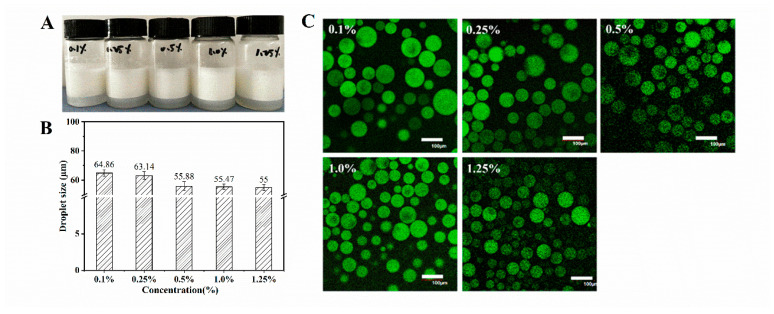
Microstructure of WPI fibrils stabilized Pickering emulsions. (**A**) Appearance, (**B**) average droplet size, and (**C**) confocal microscopic images of Pickering emulsion with different fibrils concentrations (0.1–1.25 wt.%) at pH 2.0. The scale bar was 100 μm.

The visual observation of freshly prepared Pickering emulsions at different concentrations is shown in Figure 4A. The WPI fibrils with various concentrations stabilized the Pickering emulsions and exhibited obvious creaming. The droplet sizes of the emulsions stabilized by WPI fibrils, with a fixed oil-phase fraction of 0.5, are presented in Figure 4B. The droplet size decreased with increasing WPI fibril concentration (from 0.1 wt.% to 0.5 wt.%), and the average droplet size remained relatively constant when the WPI fibril concentration increased from 0.5 wt.% to 1.25 wt.%. This suggested that there was a minimal concentration (0.5 wt.%) for the formation of homogenous WPI fibril-stabilized Pickering emulsions. CLSM was used to observe the morphology and interface properties of Pickering emulsions. The CLSM images of WPI-stabilized Pickering emulsions, with different concentrations and a fixed oil-phase fraction of 0.5, are shown in Figure 4C. WPI fibrils were labeled with ThT, which can only bind with fibrillar structures, and bright green fluorescence was observed at various concentrations, indicating all WPI fibrils were able to absorb at the oil–water interface [35]. In addition, when the concentration was low (<0.5 wt.%), the image showed relatively large emulsion droplets, which may be due to insufficient coverage of the surface of the emulsion droplets by WPI fibrils [10]. When the WPI fibril concentration increased to 0.5 wt.%, the droplet size became smaller and homogenous. This suggests the surface of droplets was sufficiently covered by fibrils at high concentrations (from 0.5 wt.% to 1.25 wt.%), and the emulsion became stable due to the electrostatic or/and steric repulsions [37]. Thus, when the WPI concentration was lower than C*, single-molecule chains existed in isolation with a low amount of WPI fibrils. When the WPI fibril concentration was greater than C*, the viscosity of the system increased due to a high degree of polymer entanglement [26], which could hinder the movement and collision of droplets.

### 3.5. Physicochemical Stability of WPI Fibril-Stabilized Pickering Emulsions

**Figure 5:**
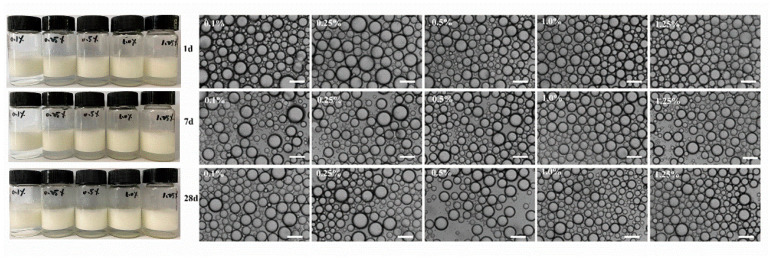
Appearance and corresponding microstructure of WPI fibrils stabilized Pickering emulsions when stored at 1 day, 7 days and 28 days. The scale bar was 100 μm.

**Figure 6:**
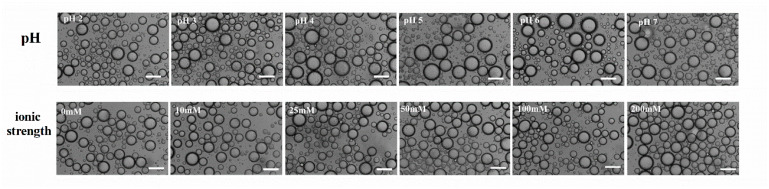
Microstructure of WPI fibrils-stabilized emulsions under different pH values (pH 2.0–7.0) and different ionic strength (0–200 mM NaCl). The scale bar was 100 μm.

Figure 5 illustrates the appearance and microstructure of the Pickering emulsion stabilized by WPI fibrils (0.1–1.25 wt.%, *v*/*v*) at pH 2 during storage for up to 28 d. The appearance of the samples did not change significantly when the emulsions were stored for 28 d. Additionally, the droplet was still stable without aggregation when the WPI fibril concentration was low (0.1 wt.%), suggesting that WPI fibrils are outstanding emulsifiers. Figure 6 shows the emulsion microstructures at various pH values and ionic strengths. No strong aggregation or droplet collapse was observed, which indicated that highly stable emulsion droplets were formed [38]. A slight increase in emulsion droplet size was observed when the pH was closer to the isoelectric point (pI), which was possibly due to a decrease in the electrostatic repulsion force at pH closer to the pI [39,40].

### 3.6. Characterization of Nobiletin-Loaded WPI Fibrils Stabilized Pickering Emulsion

**Figure 7:**
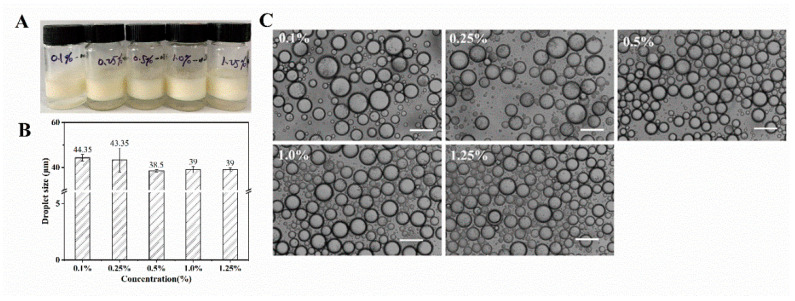
WPI fibrils-stabilized Pickering emulsion loaded with nobiletin at different fibrils concentrations (0.1–1.25%) (**A**) Appearance, (**B**) average droplet size, and (**C**) microstructure. The scale bar was 100 μm.

Figure 7 illustrates the visual appearance, microstructure, and average droplet size of the Pickering emulsion stabilized by WPI fibrils (0.1–1.25 wt.%) at pH 2 after loading 0.5 wt.% nobiletin. The emulsion droplets presented structural integrity without droplet collapse, and there was no obvious droplet coalescence. The results suggest WPI fibrils functioned well as Pickering emulsifiers to deliver nobiletin. The average droplet size of the Pickering emulsion decreased from 44.4 μm to 38.5 μm when the concentration of WPI fibril increased from 0.1 wt.% to 0.5 wt.%, and the droplet size became constant at around 39 μm, with increasing WPI fibril concentration. When the concentration of WPI fibrils was low (0.1 wt.% and 0.25 wt.%), the amount of WPI fibrils was insufficient to ensure particle surface coverage, and a smaller interfacial area was needed to prevent the coalescence of droplets. To decrease the interfacial area, the droplet size must increase [41]. When increasing the concentration of WPI fibrils to 0.5 wt.%, the sufficient coverage of WPI fibrils can facilitate the occurrence of smaller emulsion droplets with a larger surface area.

### 3.7. Lipolysis and Bioaccessibility of Nobiletin in WPI Fibril-Stabilized Pickering Emulsions

**Figure 8:**
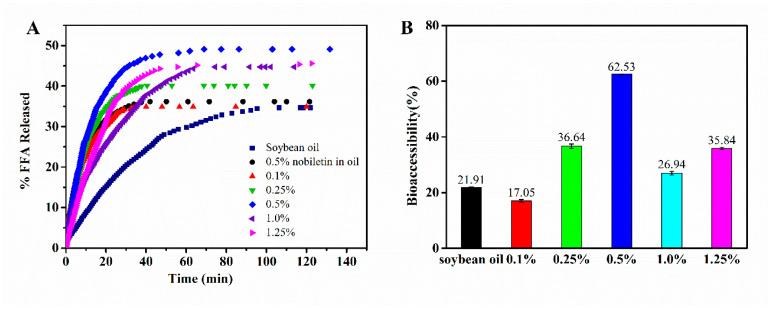
In vitro digestion of Pickering emulsion (**A**) Release profile of free fatty acids (**FFA**) and (**B**) bioaccessibility of nobiletin in soybean oil and Pickering emulsion stabilized by WPI fibrils after in vitro digestion.

Hydrophobic compounds have higher solubility in lipids and can be incorporated into the micelle core and then absorbed through the intestinal lining when lipids are hydrolyzed by lipase and micellized with bile salts, which can make the hydrophobic component become bioaccessible [28]. The in vitro lipolysis model is a useful tool to evaluate the lipid digestion kinetics and the bioaccessibility of target compounds in the delivery system [28]. During lipid digestion, pH will decrease due to the continuous release of fatty acids. To maintain the optimum pH for enzymatic digestion, sodium hydroxide was added to the digestion buffer [42]. In this study, the rate of lipolysis was determined by monitoring the volume of 0.1 M sodium hydroxide solution. Figure 8A shows the total amount of **FFA**s released from WPI fibril Pickering emulsion and soybean oil. During lipid digestion, all samples were digested rapidly in the initial stage (10–30 min), which indicated that lipase could access the emulsified lipids and catalyze the conversion of triacylglycerols into **FFA** and monoacylglycerols [43]. However, there were some differences between the extent of digestion and the digestion rates of Pickering emulsion and soybean oil. The **FFA** released from Pickering emulsions stabilized by all concentrations of WPI fibrils was significantly higher than that in soybean oil, except for a concentration of 0.1 wt.% WPI fibrils. The more **FFA** released, the higher the degree of lipolysis. Since lipolysis is an interfacial process and WPI fibril-stabilized Pickering emulsion droplets showed a larger interfacial area with a small droplet size, the Pickering emulsion had more opportunity to come in contact with the digestive enzymes [44]. In addition, the interface composition of the Pickering emulsion plays an important role in lipid digestion [45]. As shown in Figure 8B, the bioaccessibility of nobiletin in the Pickering emulsion with 0.5 wt.% WPI fibril was 62.53% ± 0.19%, which was much higher than that in soybean oil (21.91% ± 0.10%). It suggested that the higher the degree of lipid digestion, the more micelles are formed, and nobiletin will enter the micelles and be absorbed effectively.

**Figure 9:**
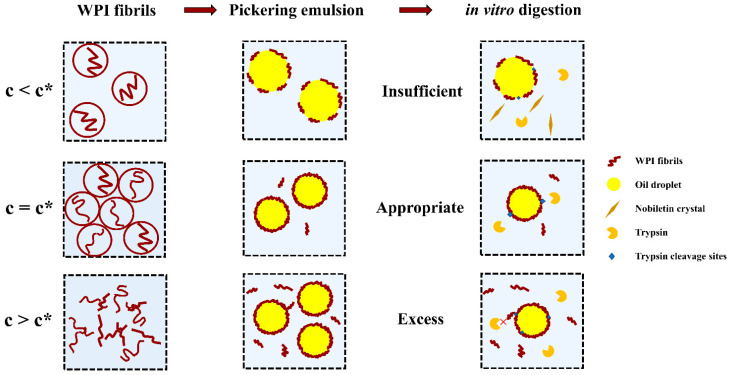
Schematic diagram of influence of WPI fibrils concentration on the emulsion structure and the bioaccessibility of nobiletin.

The effect of WPI fibrils concentration on the interfacial structure of emulsions and the bioaccessibility of nobiletin is depicted in a schematic diagram (Figure 9). When the concentration is less than C*, WPI fibrils move freely in the aqueous solution and have a high steric probability of adsorption to the oil-water interface. However, the amount of WPI fibrils is not enough to cover the full interface, resulting in large droplet size and uneven distribution of Pickering emulsions. When the concentration is close to C*, the oil droplets can be effectively stabilized, and the emulsion exhibited homogenous at an appropriate concentration necessary for full surface coverage. If continued to increase WPI fibrils, the fibrils penetrated each other and became aggregated and entangled on the surface of oil droplets. The flocculation or aggregates in emulsions may slow their rate of lipid digestion because the floc or aggregates would prevent lipase molecules from reaching the lipid droplets [46]. The in vitro bioaccessibility of nobiletin was mainly affected by the degree of lipolysis. After the lipid is digested, nobiletin enters the micelle’s structure along with it and is further absorbed by the small intestinal epithelial cells. When the interfacial structure is dense (c~C*) or there are still numerous fibrils existing in the aqueous phase (c > C*), un-adsorbed fibrils might exert a depletion effect and make Pickering emulsions have less opportunity to come in contact with the digestive enzymes.

## 4. Conclusions

In conclusion, the overlap concentration (C*) of WPI fibrils was around 0.5 wt.%. The interfacial absorption ability was improved with the concentration increasing. WPI fibrils at various concentrations can effectively stabilize Pickering emulsion and showed a long-term stability at room temperature. At C* of WPI fibrils, the droplet size of the Pickering emulsion became homogenous, and the emulsion was stable at various pHs and ionic strengths. WPI fibrils stabilized Pickering emulsion could significantly improve the degree of lipolysis and the bioaccessibility of nobiletin, especially at C* of WPI fibrils, indicating that the entanglement of WPI fibrils was an important parameter for designing food-grade Pickering emulsions. It also provides an important insight for broadening the applications of hydrophobic nutraceuticals.
